# The Mediating Role of Empathy in the Internal Mechanism of Film-Induced Tourism

**DOI:** 10.3389/fpsyg.2022.900998

**Published:** 2022-05-23

**Authors:** Kui Yi, Fengcai Li, Yanqin Zeng, Changqing Xie, Ziqi Xu

**Affiliations:** ^1^School of Business and Trade, Nanchang Institute of Science & Technology, Nanchang, China; ^2^Media Art Research Center, Jiangxi Institute of Technology of Fashion Technology, Nanchang, China; ^3^School of Economics and Management, East China Jiaotong University, Nanchang, China; ^4^Department of Art Integration, Daejin University, Pocheon, South Korea; ^5^School of Business, Foshan University, Foshan, China

**Keywords:** film-induced tourism, empathy, identity conversion, motivation transfer, demand change

## Abstract

With the advent of the information age and advancement of digital technology, film and television tourism is developing rapidly under the joint action of the film industry and tourism industry, and has become a crucial form of cultural and entertainment consumption for individuals to pursue a better life in the new age. This study designs three experiments from the perspectives of identity conversion, motivation transfer, and demand change to conduct an empirical study on the mediating role of empathy for further exploring the internal mechanism of film-induced tourism in film and television tourism. The findings suggest that the three mediation hypotheses are all valid, indicating that film-induced tourism involves identity conversion from audiences to visitors, motivation transfer from watching to traveling, and demand change from interest to expectation through emotional media.

## Introduction

As a great source of destination image information, a film effectively displays the social, cultural, and geographical characteristics of the destinations. It plays a more important role in shaping the image of destinations and inspiring the audiences’ travel motivation compared with traditional publicity media. The pictures of the films can show and convey substantial information about the destinations to the vast audiences so that they will form a preliminary impression of the destinations or change their existing cognition of the tourist destinations (Wen et al., 2018). Many tourist destinations have become popular tourist attractions through film screenings. Since the 1920s, many people have traveled to tourist destinations because they were obsessed with film stars and film scenes (Gram et al., 2020). For example, the trilogy of *The Lord of the Rings* greatly stimulated the tourism development of New Zealand between 2001 and 2003 (Cohen and Cohen, 2012). As statistics show, 6% of international tourists came to visit New Zealand on account of *The Lord of the Rings*.

With the advent of the information age and the advancement of digital technology, the boundaries of industries are shrinking or even disappearing, which promotes the industry to become a trend. In this situation, the integration of film and tourism has emerged. The combination of film and television works with tourism is a novel concept of cultural tourism (Li et al., 2021). Although tourism and film and television belong to two types of human activities, they have many common or similar characteristics. Their development has the same socioeconomic and cultural factors and plays a similar role in the development of human society (Beeton, 2016). Such a new format is called “film-included tourism” in the academic community, which intuitively reflects the close relationship between film and television and tourism (Kim et al., 2019b). Riley et al. (1998) argued that film, television, literary works, magazines, records, and videos enhance the perception of tourists, leave a profound impact and shock on the tourists, and then induce the tourists to travel to the shooting locations. According to the research of Young and Young (2008) and O’Connor et al. (2009) as screen media, film and television play similar roles in information and cultural transmission, which can exert a strong influence on the destination and promote the emergence of film and television tourism phenomenon. It is undeniable that the relationship between film and tourism has become increasingly close nowadays. Film and television are incorporating scenic spots around the world in a diversified form and presenting them in front of the audiences after beautification and processing. Empathy, as a basic function of human social activities, is the ability to experience others’ emotional and cognitive states while maintaining a unique self to understand others (Lee et al., 2017; Wang and Yi, 2020; Guthridge and Giummarra, 2021). When watching a film, the audience is attracted by the plot and scenes, thus resonating with the characters. In this case, the audiences’ emotions are amplified, and they can still retain their beliefs and attitudes in the film when returning to real-life situations.

Although more and more researchers are paying attention to the relationship between film and television and tourism, early research was mostly focused on the function of film and television tourism (Teng, 2021), the development model (Higgins-Desbiolles, 2018; Kim et al., 2019a), the existing problems and countermeasures (Yoon et al., 2015; Prayag, 2018), or business-related issues, including destination marketing (Volo and Irimiás, 2016; Beck et al., 2019), destination image (Gong and Tung, 2017), and tourist motivation (Kim and Kim, 2018). There are few studies on the internal mechanism of film-induced tourism. This study believes that, as a medium between audiences and tourist destinations, films, to some extent, make audiences experience the cognition, emotion, goal, situation, and social environment of characters through the mediating role of empathy (Grodal and Kramer, 2010). The destination image perceived through empathy with the characters attracts the audiences’ attention, provokes their travel motivation, and promotes their travel behavior, to realize the identity change from “audience” to “tourist,” promoting the development of film and television tourism.

Overall, from the perspective of the audiences, this paper explains the process of empathy as the emotional connection mechanism of the fusion between the film and tourism industries. Then, it explores the mediating role of empathy in the identity conversion from audiences to tourists, motivation transfer from film watching to traveling, and demand change from tourism interest to tourism expectation. It primarily discusses how films trigger the audiences’ emotional response which in turn affects tourism behavior to develop a deeper understanding of the internal mechanism of film-induced tourism in the hope of providing theoretical insights into the marketing practice of film and television tourism.

## Literature Review and Research Hypotheses

### Identity Conversion From Audiences to Tourists

The emotional expression of the film’s textual content and presentation of scenic spots’ images on the screen determine the identity conversion from “audiences” to “tourists” to a large extent. Through visual technology and emotional arousal, relevant information about the destinations in the film is presented and reproduced at a high level, and the positive shaping of tourist destinations can strongly strike an emotional chord with viewers (Tucker, 2016). This can change the perceptual, emotional, and intentional characteristics of the existing destination image in the minds of tourists, and create a brand-new tourist destination image (Xu et al., 2021), thereby generating tourism motivation (Hosany et al., 2020). Film works have unique advantages in spreading and shaping the positive image of tourist destinations (Gupta et al., 2020). Specifically, first, film information covers a wide range. While showing the characters, the film works will take the history, customs, food, and natural and cultural scenery of the location as the background (Beeton, 2016; Zhang et al., 2020). Second, the image of the destinations in the film works is more impressive. The film organically combines the natural landscape with the storyline, endows the scenic spots with a strong emotional color, and allows the audiences to form a deeper impression of the place where the story takes place and its culture (Albaity and Melhem, 2017; Yi et al., 2021a). In addition, the film works will bring the audience’s enjoyment of beauty. The pictures of the films are carefully designed and shot by the production team. The unique use of lenses, in combination with the 3D and other technologies (Buhalis, 2019), can discover the unique beauty of the scenery and add a dreamy cultural color to the tourist destination, thereby enhancing the audiences’ sense of familiarity and identification with the filming location (Kim et al., 2019b). The audiences put themselves into the scenario when enjoying the film, experience what the protagonist has undergone, maintain a sense of belief in the pictures after watching the film, and generate associations with the actual tourist destination to achieve the “empathy” effect (Cuff et al., 2016). Therefore, when watching a film, the audiences will feel the cognition, emotion, goal, situation, and social environment of the characters. Through their cognitive formation and emotional attachment, they identify, understand, and respond to the destination information shown in the films, generating more positive attitudes and intentions. Based on the literature review, this study makes the following hypotheses:

*H1a: The audience identity has a significant positive effect on empathy;*

*H1b: Empathy has a significant positive effect on tourist identity;*

*H1c: Empathy mediates the identity conversion from “audiences” to “tourists.”*

### Motivation Transfer From Film Watching to Traveling

As an enduring communication medium, film and television works convey the scenes and their culture to audiences in a unique way (Stam, 2017). Such transmission realizes the marketing of tourist destinations with a more realistic and intuitive effect unconsciously (Lim et al., 2021). It is undeniable that film and television works combine science and technology with cultural creativity and play an immeasurable role in the tourism market. Scholars believe that film and television works are a factor that drives audiences to travel to tourist destinations (Wen et al., 2018), which is attributed to the appeal of visual esthetics and culture. Such impetus process, just as Nordic Noir cultural practice drives Noir tourism practice, makes Northern Europe an attractive, homogenous, and exotic tourist destination, attracting numerous international tourists (Stougaard-Nielsen, 2016; Seaton, 2018; Walchester, 2018). Compared with the naked advertisement placement in the film and pure tourism promotional videos, the tourist destination appears in the film in a more appropriate way, which can attract the audiences’ attention and stimulate their travel motivation (Kim and Pan, 2018). However, Lojo et al. (2020) argued that the greater the difference between the actual tourist destination image and the ideal tourist destination image, the lower the possibility for tourists to choose this destination. Afshardoost and Eshaghi (2020) also considered that it is likely that destination image, rather than actual information, influences tourists’ choice of destination. Therefore, tourists decide on a tourist destination often based on their inner perception of the image of the tourist destination, rather than the objective facts of the tourist destination (Ryan, 2018; Yi et al., 2020). In addition, when the audiences watch a film, they often visually explore the location in the film, perceive the image of the tourist destination (Dupont et al., 2015), and draw a picture of the tourist destination in their minds, that is, passive information search (Terzidou et al., 2018). After watching a film, the audiences can actively search for information by such means as the Internet, books, interpersonal exchange, and experiences to deepen their impression and perception of the destination (Kim et al., 2017; Wang et al., 2020; Yi et al., 2021b). The performance of these behavioral characteristics just shows that film watching can stimulate the audiences’ travel motivation to trigger the travel behavior through the mediating effect of empathy. Given that, the following hypotheses are proposed:

*H2a: Visual perception has a significant positive effect on empathy;*

*H2b: Empathy has a significant positive effect on travel motivation;*

*H2c: Empathy mediates motivation transfer from film watching to traveling.*

### Demand Change From Tourism Interest to Tourism Expectation

Film and television work not only attract audiences with captivating storylines and contagious audio-visual feasts, but also can stimulate tourists’ interest in tourism destinations appearing in the film and television works such as shooting locations and the spots where the story occurs (Gong and Tung, 2017), thus there are often phenomena, psychologically known as “empathy, sympathy, or resonance”. St-James et al. (2018) summarized previous research on tourist experience, and proposed the process of film and television tourism experience including “building,” “exploration,” and “imprinting emotion.” In other words, audiences will set up emotional resonance with the characters through watching films (Kim et al., 2019b), and will develop the idea of traveling with those characters, thus generating interest in traveling. To satisfy the desire for experience, the audiences evoke the intention to go there and formulate a more complete travel plan (Yachin, 2018). Maslow’s theory divides human needs into five hierarchies, namely physiological needs, security needs, social needs, esteem needs, and self-actualization needs (Bridgman et al., 2019). A trip can be viewed to realize the need for self-worth, and a trip without plans is the pursuit of self-worth actualization. The values presented in a high-quality film and the physical and mental experiences of the tourists are the catalysts for the transformation from tourism interest to tourism expectation. Therefore, the value of film and television works plays an important role in shaping the image of the destination and the formation of tourists’ travel intentions. We need to dig deep into the embodiment of humanistic value from film and television works, instead of merely treating films as a consumer commodity. Films with a normative system of values can connect the audiences with the tourist destination, thereby triggering emotional outbursts in the audiences, prompting them to go to the setting scene to seek the satisfaction of their desire for exploration or inner drive (de Kervenoael et al., 2020) in a view to upgrading the film and television tourism to a more advanced level. Accordingly, the following hypotheses are developed:

*H3a: Tourism interest has a significant positive effect on empathy;*

*H3b: Empathy has a significant positive effect on tourism expectation;*

*H3c: Empathy mediates demand change from tourism interest to tourism expectation.*

**FIGURE 1:**
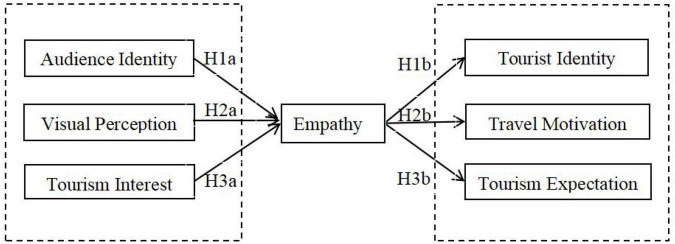
Theoretical model diagram.

Based on the above hypotheses, this study constructed a model diagram to explore the internal mechanism of film-induced tourism, that is, the mediating role of empathy, as shown in Figure 1.

## Research Design

Based on the literature review and research hypotheses, this study designed three sub-studies to verify the mediating role of “empathy” between films and tourism. Taking account of empathy, seven variables including audience identity, tourist identity, visual perception, travel motivation, tourism interest, tourism expectation, and empathy were set from the angles of identity conversion, motivation transfer, and demand change. Interviews and questionnaires were adopted to measure the psychological changes of the respondents. Respective questionnaires were designed in three sub-studies, drawing on the existing mature scales and being modified according to the interview contents and the research background. The empathy scale was designed based on the empathy scale of Tian and Robertson (2019), with a total of 10 items; the audience identity scale in sub-study 1 was based on the identity research by Umaña-Taylor et al. (2004) and Nambisan and Baron (2010), with a total of six items; the tourist identity scale was based on the role identity research by Callero (1985) and the athlete identity dimension scale by Yukhymenko-Lescroart (2014), with a total of six items; the measurement items of visual perception in sub-study 2 were based on the research on the visual cognition of the destination image by Chen et al. (2015), with a total of five items; the measurement items of travel intention were based on the scale in the research by Clark et al. (2020), which selected the motivation dimension, with a total of four items; the measurement items of interest in sub-study 3 were based on the scale of the dimensions of emotional interest and value interest in the research by Kleespies et al. (2021), with a total of six items; the measurement scale items of expectation were based on the scale in the research by Mao et al. (2010), with a total of four items. All scales in the three questionnaires adopted a 5-point Likert scale, where “1” stands for “strongly disagree” and “5” means “strongly agree.”

## Sub-Study 1: Identity Conversion From Audiences to Tourists

To verify H1c, the mediating role of empathy in the identity conversion from “audiences” to “tourists,” sub-study 1 selected the film *Lost in Thailand* as the research background, which is not only a successful comedy but also a tourist scenery film with strong tourist elements (Mostafanezhad and Promburom, 2018), reflecting the perfect match between films and tourism. It broke the box office record of local Chinese films, and stimulated the development of Thailand’s tourism industry. The film incorporated the iconic cultural elements of Thailand, showing Thailand’s folk culture and mountain scenery, and arousing the audiences’ love for the scenery in the film. When watching the film, the audiences are guided by it to virtually “tour” tourist attractions. They are often immersed in the film, evoking emotional resonance and association of being in Thailand. After watching the film, the impressive pictures will prompt the audiences to trigger tourism motives and feel like traveling with the characters, thus facilitating the identity conversion from “audiences” to “tourists.”

### Methodology

Sub-study 1 collected online data, distributed, and recovered questionnaires through an online professional research platform, and adopted the PLS-SEM method to conduct a small-sample empirical analysis. Before designing the questionnaire, the items that are appropriate to the research background were ensured. This study randomly selected 10 participants (having enough free time, willing to participate in the experiment, and being given a reward) at the entrance of a supermarket, and organized them to watch the film *Lost in Thailand* (the participants did not watch this film before). Their facial expression changes were recorded while watching the film, and they were interviewed after watching the film to record their emotional changes. The interview results found that during the film-watching process, the participants would put themselves into the film and feel like traveling with the characters. Gradually, such desire became stronger, and they would imagine themselves as tourists and evoke travel motivation.

Based on the interview results, sub-study 1 designed and distributed the questionnaires, and only the participants who had watched the film *Lost in Thailand* were eligible to participate in the study. To ensure the validity of the questionnaires, the questionnaires submitted within a too short time or with obvious contradictory answers were eliminated. In the end, a total of 85 questionnaires were recovered, 77 valid questionnaires were retained, and the questionnaire effective rate was 90.59%. Among the overall valid samples of the study, male participants accounted for 44.2% and female participants accounted for 55.8%, primarily aged 18–30 years old (accounting for 62.3% of the total), and most of them were full-time students (accounting for 49.4% of the total). The questionnaire also covered other age groups, educational backgrounds, occupations, and monthly income distribution to ensure the authenticity of the sample information.

### Findings

The PLS-SEM analysis results showed that the distribution of each dimension factor was in line with the expected settings of the scale. To ensure the reliability of the data, the required indexes for the items were all above 0.6. After excluding the non-conforming items, all the required three indexes received preferable feedback. The prediction performance of the model is shown in Table 1. In the model testing process, the R^2^ value of tourist identity was 0.542 and its adjusted R^2^ value was 0.535; the R^2^ value of empathy was 0.197 and the adjusted R^2^ value was 0.186, indicating that the latent variables had a relatively strong ability to explain tourist identity and empathy; and the model NFI (normed fit index) was 0.703, indicating that the model fit of sub-study 1 was satisfactory.

**TABLE 1:** Prediction performance of the model.

	R^2^	Adjusted R^2^
Tourist identity	0.542	0.535
Empathy	0.197	0.186

In terms of construct reliability and validity, the standardized results were adopted for analysis as shown in Table 2. As for tourist identity, Cronbach’s alpha was 0.709, rho_A was 0.714, composite reliability (CR) was 0.810, and average variance extracted (AVE) was 0.462; as for visitor identity, Cronbach’s alpha was 0.865, rho_A was 0.883, CR was 0.908, and AVE was 0.712; as for empathy, Cronbach’s alpha was 0.798, rho_A was 0.800, CR was 0.862, and AVE was 0.556. The Cronbach’s alpha of each latent variable was greater than 0.7, indicating that each latent variable had strong reliability; the CR was greater than 0.8, further proving that the model had high reliability; and the AVE of each latent variable was between 0.35 and 0.5, all in the tolerance interval. The above analysis suggested that the overall model fit was satisfactory, the internal latent relationships had a significant explanatory performance, the estimate effect was acceptable, and the reliability indices were consistent with the construct validity.

**TABLE 2:** Reliability and validity of the model constructs.

	Cronbach’s alpha	rho_A	CR	AVE
Tourist identity	0.709	0.714	0.810	0.462
Audience identity	0.865	0.883	0.908	0.712
Empathy	0.798	0.800	0.862	0.556

The Bootstrapping method was adopted to calculate the T-statistic of each path coefficient to test the significance level of the path coefficient estimate (two-tailed test), as shown in Table 3. The T-statistic of the structural equation model in the Bootstrapping test showed that all the path coefficients had high T-statistics, specifically, the T-statistic of audience identity and empathy was 1,966 (ranging from 1.96 to 2.58), and the T-statistic of empathy and tourist identity was 11.288 (>3.29), the *p*-value of each path was less than 0.05, indicating that each path coefficient passed the test of the corresponding significance level, thus the model had stable structure.

**TABLE 3:** Bootstrapping: significance test results of pathway coefficients.

	Original sample (O)	Sample mean (M)	Standard deviation (STDEV)	T statistic (| O/STDEV|)	*P*-value
Audience identity -> Empathy	0.444	0.487	0.226	1.966	0.049
Empathy -> Tourist identity	0.736	0.742	0.065	11.288	0.000

### Discussion

The above results suggested that audience identity had a significant positive effect on empathy, with a path coefficient of 0.444; besides, empathy had a significant positive effect on tourist identity, with a path coefficient of 0.736. That means, the plot and content of the film can arouse the audience’s emotional resonance, thus generating associations with tourist destinations, as if being in the tourist attractions in the film, thus empathy was fulfilled. To have a deeper experience of the joys and sorrows of the characters in the film and have a zero-distance immersion in the cultural atmosphere and unique landscape of the shooting locations, the audiences tend to change their identities and experience the cultural scene of the destinations as a tourist. The results of sub-study 1 verified that empathy plays a positive mediating role in the conversion of audience identity to tourist identity.

## Sub-Study 2: Motivation Transfer From Film Watching to Traveling

Sub-study 2 selected the Japanese anime *Your Name* as the research background to verify the mediating role of empathy in motivation transfer from film watching to traveling. In recent years, the concept, technology, and content of visual culture have developed rapidly, and animation films can especially provide individuals with a visual feast (Xu et al., 2022). A growing number of animation filmmakers are inserting reality into their works, incorporating specific local scenery into the background of the story, and more animation fans travel to the popular locations appearing in the animation works. The anime *Your Name* involves abundant shooting locations and has become one of the most popular films to visit holy places. As a result, visual esthetics has gradually become a belief in films. The audiences will have empathy with the characters in the films, which will stimulate their visual perception and emotional perception of the tourist destination. In this way, they will generate travel motivation and promote travel behavior.

### Methodology

Sub-study 2 adopted the methods of interviews and questionnaires and conducted a PLS-SEM small-sample empirical analysis of the recovered data. Like sub-study 1, sub-study 2 also randomly selected 10 participants to watch the film *Your Name* (who did not watch the film before) and recorded their facial expressions and emotional changes. The interview results suggested that the film would bring the audiences a visual impact, and the delicate emotional portrayal and realistic picture style of the film made the audiences empathize with the film. The audiences were eager to obtain visual and intuitive “confirmation” from the scenes of the holy places and thus evoke the travel motivation.

Based on the interview results, sub-study 2 designed and distributed the questionnaires, and only the participants who had watched the film *Your Name* were eligible to participate in the study. To ensure the validity of the questionnaires, the questionnaires submitted within a too short time or with obvious contradictory replies were eliminated. In the end, a total of 82 questionnaires were collected, among which 76 valid questionnaires were retained, and the questionnaire effective rate was 92.68%. In the overall valid samples of this study, male participants accounted for 44.7% and female participants accounted for 55.3%, mainly aged 18–30 years old (accounting for 67.1%), and most of them had high school education or above (accounting for 92.1%). The participants also included other age groups, educational backgrounds, occupations, and monthly income distributions to ensure the authenticity of the sample information.

### Findings

The PLS-SEM analysis results suggested that the distribution of each dimension factor conformed to the expected settings of the scale. To ensure the reliability of the data, the required indexes for the items were above 0.6. After eliminating the non-conforming items, the required three indexes obtained preferable feedback. The prediction performance of the model is shown in Table 4. In the model testing process, the R^2^ value of travel was 0.380 and the adjusted R^2^ value was 0.371; the R^2^ value of empathy was 0.584 and the adjusted R^2^ value was 0.578, indicating that each latent variable had high explanatory performance toward travel and empathy. And the model NFI was 0.649, indicating that the model fit in sub-study 2 was satisfactory.

**TABLE 4:** Prediction performance of the model.

	R^2^	Adjusted R^2^
Travel	0.380	0.371
Empathy	0.584	0.578

In terms of construct reliability and validity, the standardized results were adopted for analysis, as shown in Table 5. As for travel, the Cronbach’s alpha was 0.861, rho_A was 0.872, CR was 0.906, and AVE was 0.707; as for film watching, the Cronbach’s alpha was 0.897, rho_A was 0.898, the CR was 0.924, and the AVE was 0.708; as for empathy, the Cronbach’s alpha was 0.810, rho_A was 0.829, CR was 0.863, and AVE was 0.515. The Cronbach’s alpha coefficient of each latent variable was greater than 0.8, indicating that each latent variable had high reliability; the CR was greater than 0.8, further proving that the model had high reliability; and the AVE of each latent variable was greater than 0.5. The above analysis showed that the model had a satisfactory overall good fit, the internal latent relationship had a significant explanatory performance, the estimate effect was acceptable, and the reliability indices were consistent with the construct validity.

**TABLE 5:** Reliability and validity of the model constructs.

	Cronbach’s alpha	rho_A	CR	AVE
Travel	0.861	0.872	0.906	0.707
Film watching	0.897	0.898	0.924	0.708
Empathy	0.810	0.829	0.863	0.515

The Bootstrapping method was adopted to calculate the T-statistic of each path coefficient to test the significance level of the path coefficient estimate (two-tailed test) as shown in Table 6. The T-statistic of the structural equation model in the Bootstrapping test showed that all path coefficients had high T-statistic. Among them, if 1.96 < T < 2.58, the path coefficient was significant at the 0.05 level; if 2.58 < T < 3.29, the path coefficient was estimated to be significant at the 0.01 level; if T > 3.29, the path coefficient was significant at the 0.001 level. Specifically, the path coefficient of film watching and empathy was 18.333, and the path coefficient of empathy and travel was 4.556. The *p-*value of each path was less than 0.001, indicating that each path coefficient passed the test of the corresponding significance level, and thus the model had a stable structure.

**TABLE 6:** Bootstrapping: significance test results of pathway coefficients.

	Original sample (O)	Sample mean (M)	Standard deviation (STDEV)	T statistic (| O/STDEV|)	*P*-value
Film watching- > Empathy	0.764	0.779	0.042	18.333	0.000
Empathy - > Travel	0.616	0.625	0.135	4.556	0.000

### Discussion

The above results suggested that visual perception had a significant positive effect on empathy, with a path coefficient of 0.764; besides, empathy had a significant positive effect on travel motivation, with a path coefficient of 0.616. That is to say when the audiences are watching the film, the realistic scenery and delicate sentiment of the film will make the audiences perceive the image characteristics of the destination and generate emotional resonance so that the audiences yearn for the image of the destination and stimulate motivation to travel to the destinations. The results of sub-study 2 confirmed that empathy plays a positive mediating role in the motivation transfer from film watching to traveling to the destinations.

## Sub-Study 3: Demand Change From Tourism Interest to Tourism Expectation

To deeply explore the internal mechanism of film-induced tourism, sub-study 3 selected the series of the film *Detective Chinatown* as the research background to verify the mediating role of empathy in the demand change from tourism interest to tourism expectation. The serial *Detective Chinatown* was released from 2015 to 2021, a typical product of the new media era, which chose different countries as the settings for different series, and integrated the iconic scenic spots and culture of various countries while maintaining the same theme. In addition, each of the series involved a Chinatown and displayed the local customs and features based on the story of “Chinatown and detective” while integrating local cultural elements. The creation of the film made the audiences evoke a strong interest in the local customs, immersed in the film, and resonate with the excellent values and culture presented in the film. Moreover, the audiences who pursue high experience tend to turn their interest to expectation, and they expect to go to the shooting locations to experience the local customs and practices to meet their higher-level needs (Gao et al., 2020).

### Methodology

Like sub-study 1 and sub-study 2, sub-study 3 adopted the methods of interviews and questionnaires and conducted a PLS-SEM small-sample empirical analysis on the recovered data. However, sub-study 3 randomly asked the participants to watch different series of the *Chinatown Detective* and recorded their facial expressions and emotional changes. The interview results revealed that what appealed to the audiences was not only the suspense of the cases but also the local customs and beautiful scenery in the films. Their emotional preferences were satisfied by the films, which in turn stimulated their expectation and yearning for the real scenes appearing in the films.

Based on the interview results, sub-study 3 designed and distributed the questionnaires, and only those participants who had watched the series of the film *Chinatown Detective* were eligible to participate in the study. To ensure the validity of the questionnaire, the questionnaires submitted within a too short time or with obvious contradictory replies were eliminated. In the end, a total of 83 questionnaires were recovered, among which 77 valid questionnaires were retained, and the effective rate of the questionnaire was 92.77%. In the overall valid samples of this study, male participants accounted for 44.2% and female participants accounted for 55.8%, mainly aged 18–30 years old (accounting for 68.8%), and most of them had high school education or above (accounting for 89.6%). The participants also involved other age groups, educational backgrounds, occupations, and monthly income distributions to ensure the authenticity of the sample information.

### Findings

The PLS-SEM analysis results suggested that the distribution of each dimension factor conformed to the expected settings of the scale. To ensure the reliability of the data, the required indices of the items were all above 0.6. After eliminating the non-conforming items, the required three indexes obtained favorable feedback. The prediction performance of the model is shown in Table 7. In the model testing process, the R^2^ value of tourism expectation was 0.465 and its adjusted R^2^ value was 0.458; the R^2^ value of empathy was 0.684 and its adjusted R^2^ value was 0.680, indicating that each latent variable had high explanatory performance toward tourism expectation and empathy. The model NFI was 0.709 indicating that the model fit in sub-study 3 was satisfactory.

**TABLE 7:** Prediction performance of the model.

	R^2^	Adjusted R^2^
Tourism expectation	0.465	0.458
Empathy	0.684	0.680

In terms of construct reliability and validity, the standardized results were adopted for analysis, as shown in Table 8. As for tourism expectation, the Cronbach’s alpha was 0.920, rho_A was 0.921, CR was 0.944, and AVE was 0.808; as for empathy, the Cronbach’s alpha was 905, rho_A was 0.911, CR was 0.922, and AVE was 0.569; as for film watching interest, the Cronbach’s alpha was 0.890, rho_A was 0.898, CR was 0.916, and AVE was 0.647. The Cronbach’s alpha coefficient of each latent variable was greater than 0.8, indicating that each latent variable had high reliability; the CR was greater than 0.8, further proving that the model had high reliability; and the AVE of each latent variable was greater than 0.5. The above analysis revealed that the overall model fit was satisfactory, the internal latent relationship had a significant explanatory performance, the estimate effect was acceptable, and the reliability indices were consistent with the construct validity.

**TABLE 8:** Reliability and validity of the model constructs.

	Cronbach’s alpha	rho_A	CR	AVE
Tourism expectation	0.920	0.921	0.944	0.808
Empathy	0.905	0.911	0.922	0.569
Film watching interest	0.890	0.898	0.916	0.647

The Bootstrapping method was adopted to calculate the T-statistic of each path coefficient to test the significance level of the path coefficient estimate (two-tailed test) as shown in Table 9. The T-statistic of the structural equation model in the Bootstrapping test showed that all path coefficients had high T-statistic. Among them, if 1.96 < T < 2.58, the path coefficient was significant at the 0.05 level; if 2.58 < T < 3.29, the path coefficient was estimated to be significant at the 0.01 level; if T > 3.29, the path coefficient was significant at the 0.001 level. Specifically, the path coefficient between empathy and travel expectation was 9.592, the path coefficient between film watching interest and empathy was 19.163, thus the *p*-value of each path was less than 0.001, indicating that each path coefficient passed the test of the corresponding significance level, and the model had a stable structure.

**TABLE 9:** Bootstrapping: significance test results of pathway coefficients.

	Original sample (O)	Sample mean (M)	Standardized deviation (STDEV)	T statistic (| O/STDEV|)	*P*-value
Empathy -> Tourism expectation	0.682	0.685	0.071	9.592	0.000
Film watching interest -> Empathy	0.827	0.829	0.043	19.163	0.000

### Discussion

The above results revealed that interest had a significant positive effect on empathy, with a path coefficient of 0.682; also, empathy had a significant positive effect on expectation, with a path coefficient of 0.827. That is to say when the audiences are watching the film, the unique plot and the profound culture of the film attract their interest. For the audiences with high emotional identity, the unmet needs in their hearts are first satisfied by the film through emotional resonance with the characters and objects in the film, and the film-watching interest gradually shifts to travel expectation, resulting in the desire to travel and the need to enhance their self-worth. The results in sub-study 3 validated that empathy plays a positive mediating role in the shift from tourism interest to tourism expectation.

## Research Conclusion

### Discussion

As a new type of business that integrates the traditional film and television industry and the tourism industry, film and television tourism has developed rapidly under the joint action of these two industries and has played an essential part for individuals to pursue a better life in the new era. When an excellent tourist scenery, film attracts audiences to the screen and a charming landscape on the screen deeply impresses the audiences. A new chain is formed between the film culture and tourism industry. It has contributed to the consensus and common progress of the two industries in marketing. How film and television work promotes the development of tourism, how it transforms the “audiences” into “tourists,” how it stimulates the travel motivation of the audiences, and how it arouses the audiences’ interest in travel give rise to their demand change for travel expectation—all these questions require in-depth exploration of the internal mechanism. Therefore, this study introduced empathy as a mediating variable and initiated a series of sub-studies to empirically analyze the role of empathy in film-induced tourism. The results showed that empathy plays a decisive role in film-induced tourism and further clarified the internal mechanism of film-induced tourism.

Through the above analysis, this study drew three conclusions as follows. First, when audiences are watching a film, it is easy for them to evoke emotional resonance, as if they were in the setting of the film. When the audiences emotionally resonate with the contents of the film, they would like to travel to the destinations appearing in the film, facilitating the audience-to-tourist identity conversion (as shown in sub-study 1). Second, when the audiences are attracted by the image of the tourist destinations in the film, the emotional connection between the tourist destinations and the audiences will be further strengthened, thus arousing the audiences’ travel desire and motivation (as verified in sub-study 2). Furthermore, film tourism itself is an activity with a strong cultural atmosphere, in which tourists with high cultural appreciation capability are willing to participate. For the audiences with high emotional identity and pursuit, the characters and objects in the film can motivate them to take a great interest in traveling and expect to obtain the satisfaction of “breaking the disparity between the virtual and real world” from travel motivation to travel experience (as revealed in sub-study 3).

### Theoretical Implications

This study provides several theoretical implications. First, it expands the research on film and television tourism. Early research on film and television tourism mostly focused on the function of film and television tourism (Teng, 2021), the development model (Higgins-Desbiolles, 2018; Kim et al., 2019a), the existing problems, and countermeasures (Yoon et al., 2015; Prayag, 2018). There are few studies on the internal mechanism of film-induced tourism. Considering that empathy, as a basic function of human social activities and plays an indispensable role in film viewing and tourism, it is important to understand how empathy affects film-induced tourism. Therefore, this study introduces the variable “empathy” to explore the role of empathy in film-induced tourism.

Second, this study is helpful to study empathy. It examines an understudied role of empathy—the mediating role of empathy. The concept of empathy in previous studies was mostly applied in research in the fields of prosocial behavior, emotion recognition, and nursing (Juhl et al., 2020; Levett-Jones and Cant, 2020; Brett and Maybery, 2021), mostly on the mechanism of empathy (Clarke et al., 2015; Shin, 2018; Adriaense et al., 2020), and there are few studies on the role of empathy and its effects. The current study has taken empathy as a mediating variable, having expounded and verified the positive effect of empathy from the perspectives of identity conversion, motivation transfer, and demand change, which has enriched the theory of empathy.

Finally, this study explores the internal mechanism of film-induced tourism. This study found that in previous research on film and television tourism, scholars had discussed the mechanism of film-induced tourism from identity conversion (Kantarci et al., 2017; Du et al., 2020), motivation transfer (Rittichainuwat and Rattanaphinanchai, 2015; Hetland et al., 2016; Oviedo-García et al., 2016), and demand change (Kim and Kim, 2018; Qiang et al., 2019; Teng, 2021), however, the research mostly stayed at the basic stage and did not deeply explore its internal mechanism. This study has introduced “empathy” as the medium in which the mechanism occurs and has profoundly expounded that “empathy” as a medium can promote the audience-to-tourist identity change, film watching-to-traveling motivation transfer, and interest-to-expectation demand change, providing profound insights into the development of film and television tourism.

### Practical Implications

Films shall be endowed with more emotional and cultural connotations. Film and television tourism begins with film and television works whose appeal is the premise of film and television tourism. High-quality film and television contents and excellent value dissemination will meet individuals’ moral and cultural needs. Human beings are perceptual, and the tourists who travel because of watching the films generally have a high degree of emotional identity and cultural pursuit. Therefore, the film and television shooting must not only integrate a favorable natural environment and local customs and practices but also further understand the role of individual emotional resources in film-induced tourism so that the culture and emotions conveyed by film and television works can be deeply rooted in the hearts of the audiences, thereby attracting more audiences to travel to the locations appearing in the films.

In addition, film and television marketing shall be highlighted. A brilliant tourist destination needs to find an appropriate medium to convey its image to the tourists. The film can just rely on its technical means and manifestation modes to convey the tourist destination to the audiences with a more fascinating image. Therefore, the development of tourism needs to avail films to promote the destination images. From the perspective of the distance of tourist origins, for long-distance tourists, film and television marketing should focus on highlighting the cultural differences of tourist destinations to stimulate tourists’ exploration desires. Additionally, the tourism products in surrounding areas shall be taken into account of to form economical and attractive tourism routes; and for short-distance tourist origins, the leisure and entertainment characteristics of tourism products should be effectively highlighted.

Furthermore, diversified film and television products shall be developed to better meet the tourist expectations. With the development of economy and society, popularization of experience services, and advancement of science and technology, film and television tourism products can no longer stay in the primary mode of static viewing and picture taking, but need to increase the interaction and participation of tourists and products, and enhance their immersion and experience (Zhang, 2019). Therefore, tourist destinations should design travel routes according to the films, restore film and television scenes, increase film and television performances, or allow tourists to participate in film and television shooting so that the tourists can experience the joys and sorrows of the characters in the film at zero distance, and their tourism expectations are better satisfied in a bid to promote the prosperity of the tourism industry.

### Limitations and Future Research

There are some limitations in this study, which need to be further addressed. First, when studying the mediating role of empathy in film-induced tourism, this study only considered the role of empathy in three aspects including identity conversion, motivation transfer, and demand change, ignoring other possible factors. For example, the participation of stars in films can also play a positive role in shaping tourist destinations, arousing the audiences’ sense of celebrity worship to make them empathize, and stimulate tourism motivation and tourism behavior. Therefore, future research may consider the role of celebrity worship in the formation of the audiences’ travel motivation (Yen and Croy, 2016). Second, this study only tested the Chinese audiences, not involving the Chinese audiences living abroad as well as foreign audiences. Due to the varied cultural and educational environments of countries, the audiences in different countries may have different emotions and pursuits related to the tourist destinations in the films. The mediating role of empathy may also vary among audiences in different countries. Therefore, a comparative study of Chinese tourists in overseas markets, tourists from other countries, and local tourists will help to analyze the cross-cultural differences.

## Data Availability Statement

The raw data supporting the conclusions of this article will be made available by the authors, without undue reservation.

## Ethics Statement

The studies involving human participants were reviewed and approved by School of Economics and Management, East China Jiaotong University, China. The patients/participants provided their written informed consent to participate in this study. Written informed consent was obtained from the individual(s) for the publication of any potentially identifiable images or data included in this article.

## Author Contributions

KY contributed to the empirical work, analysis of the results, and writing of the first draft. FL, YZ, CX, and ZX supported the total work of KY. CX advised the hypotheses development. All authors discussed the results and commented on the manuscript.

## Conflict of Interest

The authors declare that the research was conducted in the absence of any commercial or financial relationships that could be construed as a potential conflict of interest.

## Publisher’s Note

All claims expressed in this article are solely those of the authors and do not necessarily represent those of their affiliated organizations, or those of the publisher, the editors and the reviewers. Any product that may be evaluated in this article, or claim that may be made by its manufacturer, is not guaranteed or endorsed by the publisher.
